# Gavi Support for Typhoid Conjugate Vaccines: Moving From Global Investments to Country Introduction

**DOI:** 10.1093/cid/ciaa342

**Published:** 2020-07-29

**Authors:** Adam Soble, Zeenat Patel, Stephen Sosler, Lee Hampton, Hope Johnson

**Affiliations:** Gavi, the Vaccine Alliance, Geneva, Switzerland

**Keywords:** enteric fever, typhoid conjugate vaccine, vaccines, immunizations, global health

## Abstract

Nine years elapsed between Gavi’s investment decision to support typhoid conjugate vaccines (TCVs) in 2008 and Gavi support becoming available for countries to introduce TCV. The protracted path toward Gavi support for TCV highlights the challenges of vaccine development for lower-income countries and the importance of Gavi engagement as early as possible in product development processes to support the alignment of manufacturing, global policy, and program implementation. Early engagement would provide inputs to inform strategic vaccine investment decisions that transition more efficiently toward country implementation. Several countries have been approved for Gavi support to introduce TCV in 2019–2020. The paucity of generalizable typhoid epidemiological data in early introducing countries has reinforced the need for continued evidence generation regarding typhoid epidemiology and TCV impact. This has led to the development of guidance and tools to support country decision making for TCV introduction based on enhanced understanding of local typhoid burden and risk.

In 2000, Gavi, the Vaccine Alliance (Gavi) was created to increase access to and affordability of new and underused vaccines for people living in low-income countries through an innovative public–private partnership model comprised of governments, the private sector, United Nations agencies, and civil society. Countries are eligible to receive Gavi support based on gross national income (GNI) per capita. As GNI per capita increases, countries increasingly finance immunization program costs before transitioning from Gavi support. Between 2000 and 2018, Gavi’s support has enabled > 760 million children to be reached and prevented 13 million future deaths through increased access to immunization services [1]. In Gavi’s strategy for the 2021–2025 period, the Alliance will continue its focus on saving lives and protecting people’s health by increasing equitable and sustainable use of vaccines through support for new vaccine introductions appropriate to country context, stronger health systems, and an increased focus on unimmunized children [2]. Many of the vaccines initially supported by Gavi were already available in high-income countries. Gavi support has catalyzed the introduction of these vaccines in many low-income countries that previously lacked access to them. Potential Gavi funding for procurement of new vaccines has also helped catalyze the development of vaccines specifically for low-income countries, including the MenAfriVac and more recently, typhoid conjugate vaccine (TCV).

Since Gavi’s inception, the portfolio of vaccines it supports to meet public health needs in low-income countries has steadily grown. In 2008, Gavi adopted a process to periodically update its vaccine investments. Gavi’s Vaccine Investment Strategy (VIS) uses standardized, evidence-based criteria to comparatively assess potential vaccine investments to inform prioritization of Gavi’s resources [3]. The scope of the VIS includes vaccines for use in preventive immunization, vaccine stockpiles for emergency response, and support for evidence generation when critical knowledge gaps exist that limit Gavi’s ability to make vaccine investment decisions [4]. Typhoid vaccines were included in Gavi’s vaccine portfolio through the 2008 VIS (Figure 1). This was noteworthy as TCV was being developed primarily for use in low-income countries without a market in upper-income countries and largely dependent on the incentive of future Gavi procurement funding.

**Figure 1.**
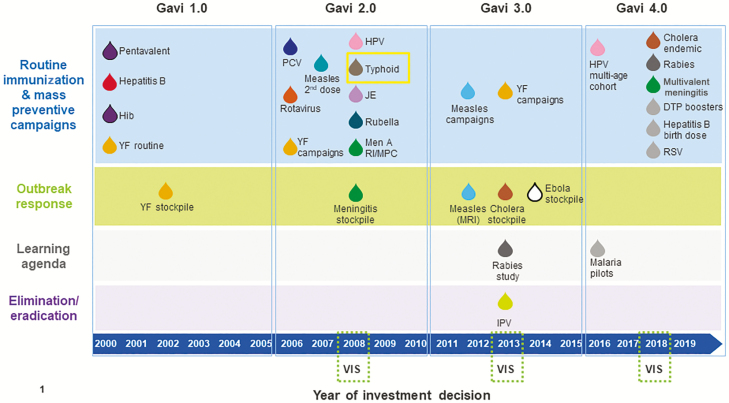
Timeline of historical Gavi investment decisions for new vaccine support, 2000–2018. Abbreviations: DTP, diphtheria, pertussis, and tetanus; Hib, *Haemophilus influenzae* type b; HPV, human papillomavirus; IPV, inactivated poliovirus vaccine; JE, japanese encephalitis; MRI, Measles & Rubella Initiative; PCV, pneumococcal conjugate vaccine; RI/MPC, routine immunisation/mass preventive campaigns; RSV, respiratory syncytial virus; VIS, Vaccine Investment Strategy; YF, yellow fever.

While Gavi’s initial investment decision to support typhoid vaccines was made in 2008, due to product development challenges and setbacks related to TCV, 9 years elapsed between Gavi’s initial investment decision and Gavi support becoming available for countries to introduce TCV. The protracted timelines for a commercially available, World Health Organization (WHO)–prequalified TCV and accompanying Gavi support for eligible countries to introduce the vaccine highlights some of the key factors related to effectively linking product development with Gavi investment decision making and implementation planning.

## HISTORY OF GAVI INVESTMENT DECISIONS FOR TYPHOID VACCINES

Gavi assessed a potential investment in typhoid vaccines in 2008. At that time, there were an estimated 16–33 million cases of typhoid fever annually, causing 216 000–600 000 deaths per year, predominantly infants and school-aged children [5]. In 2008, 2 internationally licensed vaccines existed that were shown to be safe and efficacious in individuals > 2 years of age: live attenuated *Salmonella enterica* serovar Typhi (*S*. Typhi) Ty21a strain oral vaccine administered in 3–4 doses, and Vi capsular polysaccharide (Vi-PS) injectable vaccine administered in a single dose. The Gavi Board, comprised of governments, the private sector, United Nations agencies, and civil society and responsible for Gavi’s strategic direction, policy, and investment decisions, indicated a preference to support TCV once licensed, due to its anticipated administration to infants, more robust immunologic response, and an extended duration of protection compared to Vi-PS and Ty21a. The Gavi Board chose not to make a financial commitment to TCV at that time and requested exploration of potential support for the Vi-PS vaccine for a limited duration while the regulatory requirements were completed for the lead TCV candidate. The vaccine procurement–related costs estimated for TCV were approximately US$175 million for the 2009–2015 period, assuming introduction in 14 Gavi-supported countries. The Gavi Board’s decision to prioritize TCV for inclusion in the portfolio was an important moment in Gavi vaccine investment decision making, marking the first time the Gavi Board committed to support a vaccine still under development, largely due to the inherent risks and challenges involved with vaccine development.

In 2011, the Gavi Board’s Programme and Policy Committee (PPC) was asked to approve implementation strategies for typhoid vaccines following its prioritization in the 2008 VIS. The lead TCV candidate experienced product development setbacks and the most advanced pipeline candidate was not expected to become available until at least 2016. As such, the PPC reviewed a strategy focused on the temporary use of Vi-PS through targeted one-time, wide age-range catch-up campaigns in high-burden countries until TCV became available. Ultimately, Gavi’s PPC reaffirmed a preference for TCV, thereby retaining the Board’s 2008 VIS decision due to the existence of alternative treatment options and the potential need to readminister Vi-PS because of its limited duration of protection. The product development setbacks encountered with the lead TCV candidate caused a protracted lead time between Gavi’s original investment decision and country introduction.

Between 2011 and 2017, the Bill & Melinda Gates Foundation supported efforts to accelerate the availability of TCV. This included support for multicenter hospital-based surveillance studies to improve the understanding of the regional and global burden of typhoid fever, development of updated global policy for typhoid vaccines, as well as investments to spur the development of pipeline TCV candidates. Bharat Biotech International’s Typbar-TCV was licensed in India and further evaluated through a controlled human infection model study that demonstrated 87.1% efficacy against typhoid fever in immunologically naive patients [6, 7].

In 2017, following the WHO Strategic Advisory Group of Experts on Immunization (SAGE) recommendation, Gavi updated the original analysis that informed the Gavi Board’s 2008 VIS decision to support TCV. The updated analysis found Gavi’s TCV investment would avert 0.3 deaths per 1000 fully vaccinated persons and cost US$6580 per future death averted (with a range of US$3320–US$10 420), which were comparable with existing vaccines in Gavi’s portfolio [8]. This compared with an estimated cost of US$2971 per death averted in the 2008 VIS TCV investment case, which optimistically assumed lower prices. The Gavi Board subsequently approved funding for TCV, pending WHO prequalification, with an associated US$85 million for 2019–2020 to support initial country introductions as well as US$4 million to evaluate the impact of TCV following introductions in countries eligible to receive support from Gavi. The Gavi-funded impact evaluations will enhance the global community’s understanding of TCV’s impact on typhoid fever incidence and the vaccine’s role against the spread of drug-resistant strains of *S.* Typhi and support informed country-level decision making regarding TCV introduction. WHO announced the prequalification of Typbar-TCV in January 2018, marking the first time it had prequalified a TCV. Eligible countries were able to apply for Gavi support for TCV introduction, beginning in May 2018.

## GAVI SUPPORT FOR TCV AND INITIAL COUNTRY APPLICATIONS

The support provided by Gavi to eligible countries for TCV is aligned with the 2018 SAGE recommendations and WHO position paper, which recommends prioritized introduction of TCV in countries with the highest burden of typhoid disease or a high burden of antimicrobial-resistant *S.* Typhi, through inclusion into routine immunization schedules coupled with catch-up immunization up to 15 years of age at the time of introduction, based on local epidemiology of typhoid fever, including antimicrobial resistance patterns and programmatic considerations of routine immunization programs [9]. Depending on local epidemiology of typhoid fever, countries may choose to introduce TCV in certain geographic areas. Given these epidemiological and programmatic considerations, TCV introduction decisions differ from other Gavi-supported vaccines, such as rotavirus and pneumococcal conjugate vaccine, which are recommended for universal use among all children in all countries. Unlike the rotavirus and pneumococcal conjugate vaccine programs, which were supported by demand and introduction preparation initiatives that helped prepare for vaccine introduction and supported countries in generating data for vaccine introduction decision making, such support structures were not in place for TCV when a WHO prequalified vaccine became commercially available.

Since the Gavi Board’s decision in 2017 to open a funding window to support TCV, 3 countries—Pakistan, Liberia, and Zimbabwe—have been approved to receive Gavi support to introduce TCV into their respective routine immunization systems. Pakistan became the first country to introduce TCV with support from Gavi. In November 2019, Pakistan conducted a catch-up immunization campaign and reached > 9 million children aged 9 months–15 years with TCV in high-risk urban areas of Sindh province. Pakistan’s TCV introduction into routine Expanded Programme on Immunization began in December 2019. Due to both epidemiological and vaccine supply considerations, Pakistan’s TCV introduction is being conducted in phases and the catch-up immunization is being targeted at urban areas. Liberia and Zimbabwe plan to introduce TCV in 2020. Each of the countries which have applied for Gavi support used different data to justify their vaccine introduction and vaccination strategy decisions. Pakistan and Zimbabwe’s introduction decisions were based on evidence of blood culture–confirmed typhoid either through research-oriented prospective hospital-based typhoid surveillance or passive surveillance, evidence of extensively drug-resistant or multidrug-resistant strains of *S.* Typhi outbreaks, as well as assessments of water, sanitation, and hygiene (WASH) as an indicator of potential typhoid fever risk and country-specific estimates of modeled typhoid burden. Liberia’s introduction decision was not informed by reliable, age-specific typhoid burden data based on blood culture confirmation. Instead, Liberia’s TCV introduction decision was informed by country-specific estimates of modeled typhoid burden; evidence of nontraumatic ileal perforations; and indicators of potential typhoid fever risk, including WASH and comparison with neighboring countries with similar typhoid risk factor profiles where estimates of typhoid fever incidence based on evidence of blood culture confirmation existed. Several other countries are currently in the advanced stages of decision making regarding TCV introduction, which may result in additional applications for Gavi support to be submitted in 2020.

Countries interested in potentially introducing TCV are advised to conduct a thorough assessment of domestic typhoid disease burden and risk factors to inform vaccine introduction and delivery strategies that are context specific and programmatically and financially feasible. Gavi’s current application guidelines for TCV include instructions for countries to collate, assess, and interpret past, current, and likely future typhoid risk, including laboratory-confirmed disease and risk factors for typhoid. This information must be submitted as part of country applications to inform the rationale for requesting Gavi support and a country’s selected vaccination strategy. Support from the Gavi Secretariat and Alliance partners is available to support country evaluation of evidence and application development. Country applications for TCV are reviewed by Gavi’s Independent Review Committee, in line with other Gavi supported vaccines. Applications are reviewed to determine the sufficiency of evidence justifying selected vaccination strategies as well as linkages to coverage and equity objectives, including strategies to reach populations at highest risk for typhoid.

## KEY LESSONS LEARNED FOR FUTURE VACCINE INVESTMENT DECISIONS

The Gavi Board’s commitment to support TCV though not yet commercially available was unprecedented, marking the first time the Gavi Board committed to support a vaccine still under development. At the time of the Gavi Board’s original TCV investment decision, significant product development and epidemiological uncertainties existed that influenced the structure of the Board’s decision and its delayed financial commitment until a pipeline TCV candidate neared commercial availability. Gavi has historically relied on other stakeholders to ensure a coherent pathway from product development to introduction, which necessitated much of the Bill & Melinda Gates Foundation’s effort to bring TCV to market [10]. Enhanced Gavi engagement further upstream in the product development and regulatory process could potentially help to better align manufacturing, global policy, and program implementation planning well in advance of new vaccine products nearing commercial availability and provide the necessary inputs to inform Gavi’s strategic vaccine investment decisions, which facilitate country use of vaccines. The product development delays for a WHO-prequalified TCV highlighted the risks and technical challenges, including conjugate vaccine technology, involved in the development of a new vaccine for low-income countries. In the future, it will be important for these risks to be well understood within the Gavi Alliance, including the potential for vaccine development failure or protracted timelines to country introduction.

Informed by the TCV experience, the Alliance has also evolved the ways in which vaccine policy making and implementation are designed and operationalized. For example, in the 2018 VIS, a learning agenda was developed for prioritized potential vaccine investments to address key knowledge gaps, including improved understanding of disease burden and optimal delivery strategies, that would help inform the design of future Gavi vaccine programs and country vaccine decision making. The internal structure of the Gavi Secretariat was also modified to establish closer links between market shaping, policy, and implementation to enhance Gavi decision making and program design.

## KEY LESSONS LEARNED FROM EARLY TCV PROGRAM IMPLEMENTATION

Despite improvements in understanding of the burden of typhoid fever in low-income contexts, many countries still lack surveillance data incorporating confirmation of cases through blood culture, currently recommended by WHO for diagnosis of typhoid fever, due to capacity and financial limitations, as well as the technical challenges of typhoid blood culture testing [11–13]. Therefore, country-level decision makers have limited or no reliable epidemiological data to guide the decisions for use of TCV as recommended by WHO. This has also created challenges for Gavi to design a funding window that ensures cost-effective health impact and feasible TCV use. Limited disease burden data have created uncertainty about the global demand for TCV and may have unintended consequences on the health of the TCV market, including product choice and available supply, which are dependent on manufacturer product development and investment decisions.

Although blood culture has low sensitivity for *S*. Typhi, resulting in potentially underestimation of typhoid fever incidence, the simpler Widal test that was a staple of typhoid testing in many countries has been shown more recently to be nonspecific, resulting in overestimation of typhoid fever incidence, a problem shared by currently available typhoid rapid diagnostic tests [14]. Additionally, the fraction of typhoid fever attributable to short-cycle vs long-cycle transmission has not been well established, which limits our understanding of the drivers of typhoid’s heterogenous epidemiology [15]. The reliable data produced from hospital-based sentinel surveillance sites demonstrate that transmission of *S.* Typhi is heterogenous spatially and temporally, limiting the generalizability and utility of those data in supporting the development of cost-effective vaccination strategies in settings with variable incidence outside of the areas covered by those sentinel sites. This heterogeneity in part influenced WHO’s recommendation for TCV, which indicates that countries need to select a context-appropriate vaccination strategy for TCV introduction based on factors including analysis of typhoid disease burden, risk factors for transmission, and availability of quality surveillance data, as well as operational feasibility considerations. However, from the initial implementation of Gavi’s TCV program to enable appropriate uptake of TCV, countries have demonstrated that additional guidance, tools, and support are required to make informed TCV introduction decisions using data that are readily available.

It is also important to recognize that Gavi support for TCV is occurring in a different context than historical support for other new vaccines for infants. First, as noted above, TCV does not have a WHO recommendation for universal use; as such, countries require more resources and data to inform decision making for introduction and implementation planning. Additionally, as many countries begin to transition from Gavi support and as Gavi’s portfolio of vaccine support continues to expand, the complexity of decision making for new vaccine introductions is increasing and, in many contexts, local burden of typhoid fever must be demonstrated to justify the introduction of TCV over other vaccines for which more robust data may be available. Finally, there remains a need to better understand performance of TCV in a range of settings, populations, and delivery strategies to inform decision makers at global, regional, and country levels. The global typhoid community is still in the early stages of understanding TCV’s impact on typhoid fever incidence, the vaccine’s duration of protection, and how it should be optimally used, including in response to outbreaks of typhoid fever. This contrasts with the Gavi experience with other vaccines, which had been in use for years in high-income countries before being introduced in countries eligible for Gavi support. Addressing these uncertainties will bolster global guidance and recommendations for TCV but also inform country-level decision making for TCV introduction.

In light of the diagnostic challenges related to typhoid fever and our evolving understanding of typhoid fever epidemiology, further investigation of typhoid transmission dynamics, including the role of chronic carriers and the transmissibility of drug-resistant strains, is also required to support intervention strategies and optimal use of TCV in different endemicity settings. Given the challenges and limitations of laboratory-confirmed typhoid burden data, there is a critical need to establish pragmatic ways to assess typhoid burden using existing surveillance and proxy data as well as new prospective methods including serosurveys and environmental surveillance to inform TCV introduction and delivery decisions. There is also need for better understanding of the outbreak potential of typhoid and optimal response strategies, which may include TCV, to effectively halt transmission. These issues, along with other barriers that exist for country-level TCV introduction decisions, require further understanding to enable appropriate deployment of TCV in endemic and outbreak settings.

The challenges and uncertainties that remain to be addressed to optimize Gavi’s support for TCV and ensure uptake of context-appropriate TCV vaccination strategies have also highlighted the critical role Gavi has following its investment decisions for continued learning and evaluation. It is imperative that, moving forward, investments are made in advance of Gavi program implementation to improve the understanding of key data gaps and generate critical insights that address key uncertainties to inform policy, program design, and country decision making.

The availability of TCV has ushered in a new era of typhoid control, and Gavi remains committed to supporting TCV introduction and is eager to continue collaborating with the global typhoid community to address the challenges that remain in our understanding of typhoid as a disease and the optimal use of TCV to combat it.

## Notes

***Supplement sponsorship.*** This supplement is funded with support from the Coalition against Typhoid Secretariat, housed at the Sabin Vaccine Institute in Washington, DC and made possible by a grant from the Bill & Melinda Gates Foundation.

***Potential conflicts of interest.*** The authors: No reported conflicts of interest. All authors have submitted the ICMJE Form for Disclosure of Potential Conflicts of Interest.
